# SPECT/CT Fusion in the Diagnosis of Hyperparathyroidism

**Published:** 2015

**Authors:** Yoshio Monzen, Akihisa Tamura, Hajime Okazaki, Taichi Kurose, Masayuki Kobayashi, Masatsugu Kuraoka

**Affiliations:** Department of Radiology, Hiroshima Prefectural Hospital, Hiroshima, Japan

**Keywords:** ^99m^Tc-MIBI parathyroid scintigraphy, Fusion image, Hyperparathyroidism, SPECT/CT

## Abstract

**Objective(s):**

In this study, we aimed to analyze the relationship between the diagnostic ability of fused single photon emission computed tomography/ computed tomography (SPECT/CT) images in localization of parathyroid lesions and the size of adenomas or hyperplastic glands.

**Methods:**

Five patients with primary hyperparathyroidism (PHPT) and 4 patients with secondary hyperparathyroidism (SHPT) were imaged 15 and 120 minutes after the intravenous injection of technetium99m-methoxyisobutylisonitrile (^99m^Tc-MIBI). All patients underwent surgery and 5 parathyroid adenomas and 10 hyperplastic glands were detected. Pathologic findings were correlated with imaging results.

**Results:**

The SPECT/CT fusion images were able to detect all parathyroid adenomas even with the greatest axial diameter of 0.6 cm. Planar scintigraphy and SPECT imaging could not detect parathyroid adenomas with an axial diameter of 1.0 to 1.2 cm. Four out of 10 (40%) hyperplastic parathyroid glands were diagnosed, using planar and SPECT imaging and 5 out of 10 (50%) hyperplastic parathyroid glands were localized, using SPECT/CT fusion images.

**Conclusion:**

SPECT/CT fusion imaging is a more useful tool for localization of parathyroid lesions, particularly parathyroid adenomas, in comparison with planar and or SPECT imaging.

## Introduction

Technetium-99m methoxyisobutylisonitrile (^99m^Tc-MIBI) parathyroid scintigraphy is a useful tool for localization of lesions in hyperparathyroidism (HPT). However, few reports have reviewed the diagnostic ability of single photon emission computed tomography/computed tomography (SPECT/CT) imaging in HPT using fusion images. In the present study, we aimed to evaluate the correlation between the diagnostic ability of fused SPECT/CT images in localization of parathyroid lesions and the size of adenomas or hyperplastic glands.

## Methods

Nine patients with HPT underwent MIBI using SPECT/CT imaging between May 2012 and April 2014. Five patients were diagnosed with primary hyperparathyroidism (PHPT) and 4 patients diagnosed with secondary hyperparathyroidism (SHPT) with chronic renal failure. All patients underwent surgery. Five lesions were identified as parathyroid adenomas, while 10 lesions were identified to be parathyroid hyperplasia.

Parathyroid hormone and calcium levels remained within the normal range in 8 patients after the surgery, although parathyroid hormone level increased after the surgery in 1 patient with PHPT.

The SPECT/CT equipment used in this study was Symbia T16 (Siemens, USA), which combines 16-slice multidetector CT and SPECT. Six-hundred MBq of ^99m^Tc-MIBI was injected intravenously in all patients. Early and delayed neck and upper thorax planar images were acquired 15 and 120 minutes after the injection, respectively. SPECT/CT acquisition was performed immediately after obtaining the delayed planar images; SPECT images were obtained using the SPECT/CT system. Overall, 90 projections (128×128 matrix) were acquired (20 seconds each), with a total duration of 15 minutes for the whole SPECT/CT procedure.

CT was performed immediately after SPECT imaging. The main CT parameters were 130 kV, 25 mAs, and a 1.2 mm slice thickness; no intravenous contrast medium was used. SPECT/CT data were analyzed on a Syngo workstation, which provided transaxial, coronal, and sagittal slices of SPECT, CT, and fused SPECT-CT data. The size of the lesions was obtained from the pathology reports.

### Statistical analysis

To compare the mean size of lesions in two groups, we used two-tailed student’s t-test. For statistical analysis, Excel 2012 was used (SSRI Co., Ltd., Japan). P-value less than 0.05 was considered statistically significant.

## Results

Five parathyroid adenomas and five hyperplastic glands were correctly localized, using the SPECT/CT fusion images. In contrast, 5 hyperplastic parathyroid glands could not be detected by fusion images.

**Figure 1:**
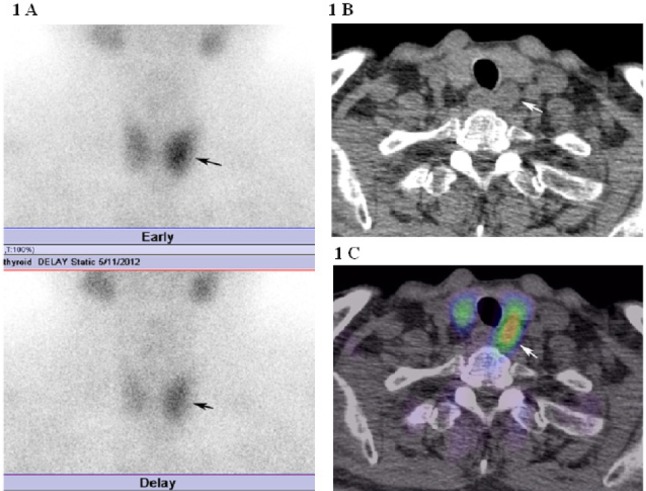
A. Initial and delayed phases of planar ^99m^Tc-MIBI scintigraphy showed increased activity in the left lobe (arrow). MIBI could not be used to differentiate between a parathyroid lesion and a thyroid lesion. B. Axial non-contrast enhanced CT of the neck. A lesion was detected posterior to the left lobe of the thyroid (arrow). C. Fusion axial image of SPECT/CT. The radiotracer accumulated in the lesion posterior to the left lobe of the thyroid (arrow). Adenoma of the left lower parathyroid gland was removed during surgery

Three out of five parathyroid adenomas were localized on ^99m^Tc-MIBI planar scintigraphy and SPECT images, whereas 5 parathyroid adenomas were localized on the SPECT/CT fusion images. All parathyroid adenomas with the largest axial diameter of 0.6 cm or more were localized on the SPECT/CT fusion images. In contrast, parathyroid adenomas with the greatest axial diameter of 1.0 to 1.2 cm were not localized on planar ^99m^Tc-MIBI scintigraphy or SPECT images (Figure 1).

The mean size of the detected and undetected parathyroid adenomas in planar scintigraphy was 1.3±0.6 and 1.1±0.1 cm, respectively (*P*=0.7). The corresponding values were 1.3±0.6 and 1.1±0.1 cm in SPECT imaging, respectively (P =0.7). The mean size of detected adenomas was 1.2±0.4 cm, based on the SPECT/CT images, and no adenoma remained undetected, using SPECT/CT images.

Four out of 10 (40%) hyperplastic parathyroid glands were localized, using planar ^99m^Tc-MIBI scintigraphy and SPECT images, while 5 out of 10 (50%) hyperplastic parathyroid glands were localized, using the SPECT/CT fusion images. Five hyperplastic parathyroid glands with the greatest axial diameter of > 0.4 cm were correctly localized, using the SPECT/CT fusion images.

The mean size of 5 hyperplastic parathyroid glands detected on SPECT/CT fusion images was 0.6±0.2 cm (range 0.4∼0.9cm). Five scintigraphically undetected hyperplastic parathyroid glands had the mean greatest axial diameter of 0.6±0.3 cm (range: 0.4-1.1 cm); there was not a statistically significant difference between the two groups (*P*=1.0). Although this negative result may reflect the small sample size in our study, it seems that other factors may play a role in the detection of hyperplastic glands, using scintigraphy (Table 1).

**Table 1:** Characteristics of the patients and detection of lesions using ^99m^Tc-MIBI planar, SPECT and SPECT/CT fusion image

Patient	Age	Sex	Pathology	Localization of the lesions	Size of lesions (cm)	Planar	SPECT	SPECT/CT
1	75	M	A	LIPG	1.0 × 3.5	No	No	Yes
2	64	F	A	LSPG	1.5 × 1.7	Yes	Yes	Yes
3	30	F	A	LIPG	0.6 × 1.4	Yes	Yes	Yes
4	62	F	A	LSPG	1.2 × 1.4	No	No	Yes
5	63	M	A	LIPG	1.7 × 1.3	Yes	Yes	Yes
6	45	F	H	RSPG	0.6 × 1.3	No	No	No
				RIPG	0.4 × 0.8	No	Yes	Yes
				LSPG	0.4 × 0.5	No	No	No
7	75	F	H	RIPG	0.5 × 1.1	Yes	Yes	Yes
				LSPG	0.7 × 1.2	Yes	Yes	Yes
8	54	M	H	RSPG	1.1 × 1.4	No	No	No
				RIPG	0.4 × 0.4	Yes	Yes	Yes
				LSPG	0.4 × 0.9	No	No	No
9	46	F	H	RIPG	0.4 × 0.6	No	No	No
				LSPG	0.9 × 1.2	Yes	No	Yes

A: Adenoma, H: Hyperplasia, M: Male, F: Female

LSPG: Left superior parathyroid gland LIPG: Left inferior parathyroid gland

RSPG: Right superior parathyroid gland RIPG: Right inferior parathyroid gland

Size of the lesion: Greatest axial diameter x cranio-caudal length

**Figure 2:**
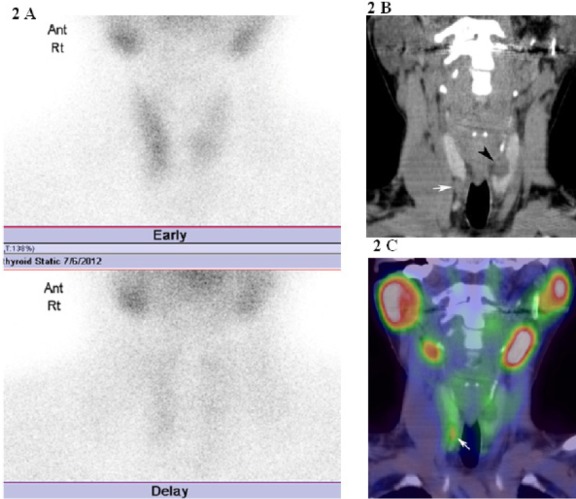
A. Initial and delayed phases of ^99m^ Tc-MIBI planar scintigraphy showed abnormal uptake. B. Coronal non-contrast enhanced CT of the neck. A lesion was detected in the inferior right portion of the thyroid lobe (arrow). A cystic mass was detected in the left lobe of the thyroid (arrowhead). C. A coronal fused image (SPECT/CT). The fusion image demonstrated a faint abnormal uptake of ^99m^Tc-MIBI in the inferior right portion of the thyroid lobe (arrow). Right inferior hyperplastic parathyroid gland was removed during surgery

Figure 1 shows a patient with PHPT due to left inferior parathyroid adenoma, which is not localized by planar scintigraphy, while detected by SPECT/CT imaging. Figure 2 shows a patient with SHPT due to right inferior hyperplastic parathyroid gland, which is not localized using planar scintigraphy, while detected by SPECT/CT imaging.

## Discussion

The main advantage of SPECT/CT systems is their application for the acquisition of high-precision fusion images, using both CT and SPECT imaging. Fusion images can be used for making a differential diagnosis between benign and malignant diseases in different body organs, determining the stage of the lesion, confirming a metastasis or its recurrence, and selecting a treatment strategy. SPECT/CT imaging can be also used to clarify the existence of an abnormal uptake, based on the diminution of scattered radiation (1, 2).

Although planar ^99m^Tc-MIBI has been used in localization of parathyroid adenomas or hyperplasia, it is not always easy to detect these lesions in the delayed phase. In this regard, Shafiei et al. investigated the diagnostic ability of ^99m^Tc-sestamibi parathyroid SPECT/CT imaging in 48 patients with PHPT. The sensitivity and specificity of SPECT/CT for localization of parathyroid adenomas were 78% and 97%, respectively. These results indicate that SPECT/CT is a useful tool for localizing parathyroid adenomas (3).

Ciappuccini et al. analyzed 94 patients with PHPT. In their study, dual-phase ^99m^Tc-sestamibi scintigraphy with SPECT/CT enabled the diagnosis of parathyroid adenomas in 56 out of 94 patients (63%) with PHPT (4). Furthermore, Papathyanassion et al. and Li et al. reported that ^99m^Tc-MIBI SPECT/CT is useful in detecting an ectopic parathyroid gland in patients with HPT (5, 6).

Meanwhile, Torregrosa et al. performed ^99m^Tc-MIBI scintigraphy in patients with PHPT (n=16) and SHPT (n=22) and found 93% and 54% sensitivities for lesion localization in cases with PHPT and SHPT, respectively. They found that ^99m^Tc-MIBI planar scintigraphy can be used as the imaging technique of choice for preoperative localization of an abnormal parathyroid gland in patients with PHPT, but only as a complementary imaging technique in patients with SHPT (7).

In addition, Caldarella et al. reviewed 24 studies on 471 patients with SHPT, using ^99m^Tc-MIBI planar images. The sensitivity and specificity of ^99m^Tc-MIBI planar scintigraphy in detecting hyperplastic glands in SHPT patients were 58% and 93%, respectively. Considering the inadequate diagnostic accuracy of this technique, it should not be considered a first-line diagnostic imaging method for preoperative localization in patients with SHPT (8).

In the present study, the detection rate of hyperplastic parathyroid glands, using ^99m^Tc-MIBI planar or fusion imaging, was poor. The degree of ^99m^Tc-MIBI uptake in parathyroid gland is influenced by the content of mitochondria-rich oxyphil cells. It is thought that ^99m^Tc-MIBI easily accumulates in parathyroid adenomas, which contain many oxyphil cells (9, 10).

In our study, the detection rate of hyperplastic gland, using ^99m^Tc-MIBI planar, SPECT, and fusion imaging was poor. Although ^99m^Tc-MIBI planar scintigraphy was not successful in the localization of hyperplastic glands, in patient No. 6, fusion images were effective in the localization of hyperplastic gland with a faint abnormal uptake of ^99m^Tc-MIBI in the right lower parathyroid (Figure 2).

No previous studies have evaluated the correlation between the diagnostic ability of fusion images and the size of parathyroid lesions or hyperplastic glands, based on pathological evaluations. In the current study, size of lesions in pathological evaluations was used for making comparisons. It showed that fusion images could detect parathyroid adenomas with the greatest axial diameter of > 0.6 cm, while planar ^99m^Tc-MIBI scintigraphy and SPECT imaging were unable to detect parathyroid adenomas with the largest axial diameter of 1.0-1.2 cm.

It can be concluded that SPECT/CT fusion imaging is a more useful tool for the localization of parathyroid lesions in hyperparathyroidism, especially parathyroid adenomas, in comparison with planar ^99m^Tc-MIBI parathyroid scintigraphy or SPECT imaging.

### Conflicts of interest

None of the authors have a direct or indirect financial interest in the products under investigation or the subject matter discussed in the article.
